# Preparation and Irreversible Inhibition Mechanism Insight into a Recombinant Kunitz Trypsin Inhibitor from *Glycine max* L. Seeds

**DOI:** 10.1007/s12010-020-03254-5

**Published:** 2020-02-01

**Authors:** Yanji Xu, Panpan Zhang, Xiao Liu, Zhike Wang, Suxia Li

**Affiliations:** 1 State Key Laboratory of Bioreactor Engineering, East China University of Science and Technology, Shanghai, 200237 China; 2 Shanghai Yaxin Biotechnology Limited Company, Shanghai, 200231 China

**Keywords:** Soybean Kunitz trypsin inhibitor, Protein refolding, Biochemical property, Inhibition kinetic assay, Molecular modeling

## Abstract

Soybean Kunitz trypsin inhibitor (SKTI), extracted from soybean (*Glycine max* L.) seeds, possesses insect resistance and anti-tumor properties. But its specific mechanisms of action are not yet known. This article reports an efficient method to produce recombinant SKTI (rSKTI) in *Escherichia coli*, reveals some biochemical properties of rSKTI, and discusses the inhibition mechanism of SKTI. The rSKTI was expressed as inclusion body in *E. coli* BL21 (DE3). After refolding, the active rSKTI was obtained and was further purified with anion-exchange chromatography (DEAE-FF) efficiently. There were similar biochemical properties between SKTI and rSKTI. The optimum pH and the optimum temperature were pH 8.0 and 35 °C, respectively, being stable during pH 7.0–11.0 and below 37 °C. The activity against trypsin was inhibited by Co^2+^, Mn^2+^, Fe^3+^, Al^3+^, and epoxy chloropropane. Inhibition kinetic assay of SKTI against trypsin as Lineweaver-Burk plots analysis both showed an unchanged *K*_m_ and a decreased *V*_max_ with *N*-benzoyl-l-arginine ethyl ester (BAEE) as substrate. Molecular modeling showed Arg63 of SKTI (active residue of SKTI) that interacts with four residues of trypsin, including three catalytic site (His57, Asp102, and Ser195) and one binding site (Asp189), forming five interactions. These provide reference for understanding the inhibition mechanism of such kind of Kunitz trypsin inhibitors.

**Electronic supplementary material:**

The online version of this article (10.1007/s12010-020-03254-5) contains supplementary material, which is available to authorized users.

## Introduction

**Fig. 1:**
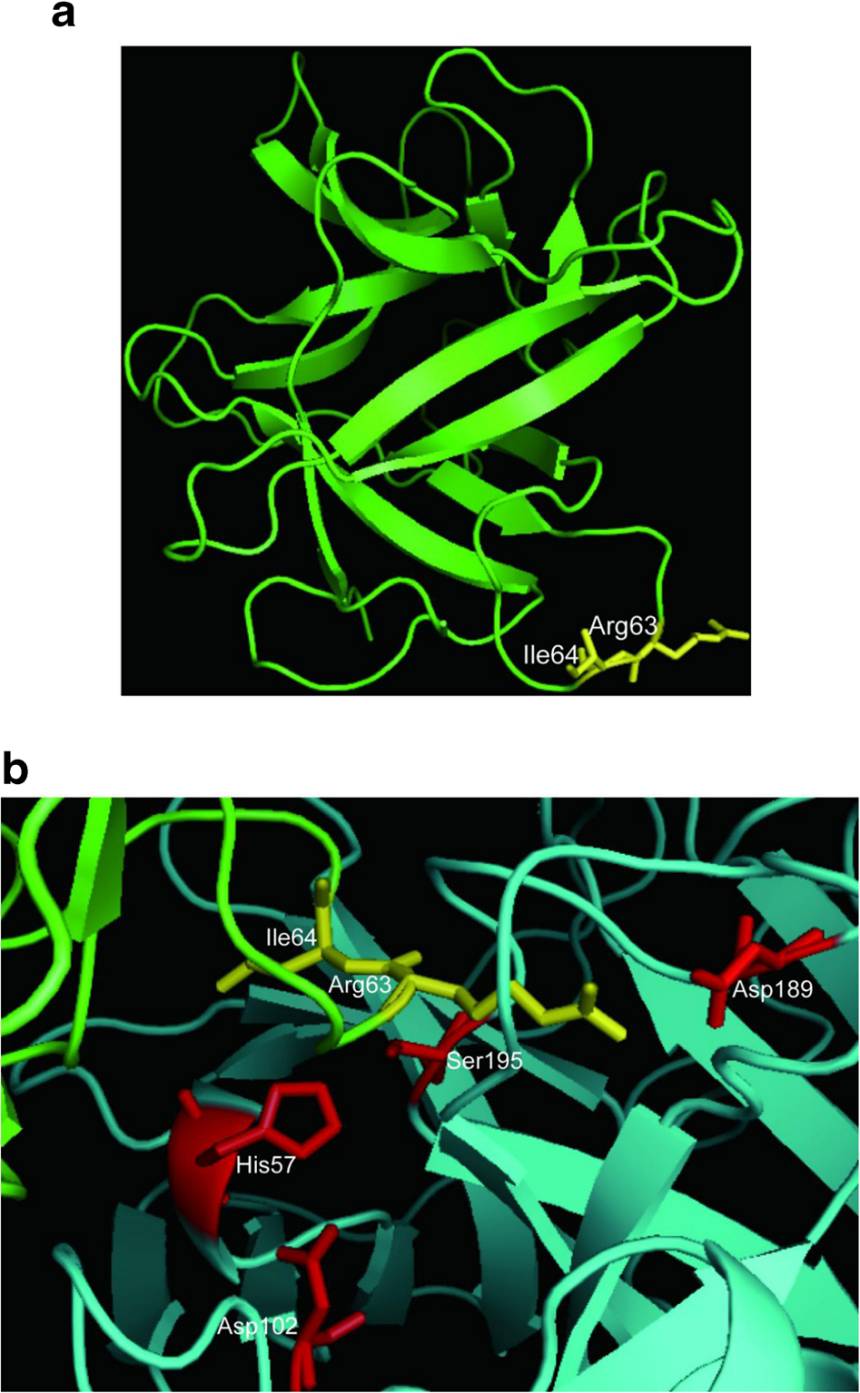
Three-dimensional model gives a general view. **a** SKTI (green) and its active sites (yellow). **b** Showing the interactions between SKTI (yellow) and trypsin (red)

Soybean (*Glycine max* L.) is one of the most important and widely consumed legume crops in the world. SKTI, a member of the serine protease inhibitor family, is a major anti-nutritional factor in soybean seeds that can inhibit the activity of both trypsin and chymotrypsin [1, 2]. These inhibitors have been implicated in various physiological functions, such as regulator of endogenous protease, storage proteins, and defense molecules against plant pests and pathogens [3]. In soybean seeds, SKTI is synthesized as a precursor of 217 amino acids that would undergo proteolytic process to remove a signal peptide of 25 amino acid residues at N terminus and a hydrophobic polypeptide of 11 amino acid residues at C terminus, yielding a mature peptide of 181 amino acids [4, 5]. The mature inhibitor is described as a low cysteine content forming two disulfide bonds. Kunitz trypsin inhibitors including SKTI have a common structure composed of 12 anti-parallel β-strands separated by irregular loops [6]. In SKTI, the side chain of Arg63 residue, as an active site residue, carried positive charges, forming strong electrostatic interaction with the negative charge of the side chain of Asp189 in enzyme, significantly contributing to the binding of inhibitor to the active center of trypsin. Figure 1 gives a whole view that the active residue Arg63 of SKTI combines with the active center of trypsin to form a stable enzyme-inhibitor complex. In this article, inhibition kinetics of SKTI to trypsin was investigated; molecular docking technology was adopted to give an explanation of the inhibition mechanism. According to a combination of inhibition kinetic behavior and molecular structure modeling, we concluded that the inhibition type should be an irreversible inhibition instead of a competitive one. This might provide reference for understand the inhibition mechanism of such kind of Kunitz trypsin inhibitors.

Trypsin inhibitors are important biochemical substances. Traditionally, SKTI was extracted from soybean seeds, which limited the large-scale application in agriculture and clinic because of the high costs of preparation [7, 8]. With the development of transgenic technology, *Escherichia coli* host has been widely used as a tool to produce various recombinant protein. Production of recombinant protein provides a suitable method for commercializing medical products [9]. Another advantage of producing recombinant proteins is better safety in comparison with sample expressed from animal cell. Perhaps considering the inhibitory ability of SKTI to serine protease, there were few reports on recombinant expression of SKTI in prokaryote [10]. Fortunately, there have been many studies about recombinant expression of SKTI in plants to harvest the resistant plants [11–14], which provided some guidance and experience for us. Here, we reported *E. coli* system was used to express rSKTI with success. In addition, the refolding conditions of rSKTI inclusion bodies were optimized. The technology would be useful for the production and study of other Kunitz trypsin inhibitors. Biochemical properties of both SKTI and rSKTI were investigated in the research, such as optimum pH and temperature, stability of pH and temperature, and inhibition kinetics behavior. Some was first studied and the results should be useful for its application.

## Materials and Methods

### Materials

The synthesis and analysis of SKTI gene sequence were performed by Generay Biotechnology Corporation (Shanghai, China). The recombinant trypsin was acquired from Yaxin Biotechnology Limited Company (Shanghai, China). The natural soybean Kunitz trypsin inhibitor (SKTI) and *N*-benzoyl-l-arginine ethyl ester (BAEE) were purchased from Sigma Co. (USA). All other reagents were of analytical grade.

### Construction of the Expression Strain for rSKTI

The gene of SKTI was designed according to the codon bias of *E. coli* and synthesized based on the primary sequence of SKTI from Uniprot database with accession number of P01070. The gene was cloned into pET-28a (+) expression vector (Novagen) using the *NdeI* (upstream) and *HindIII* (downstream) cloning sites and then transformed into *E. coli* (DE3) strains which was held in our laboratory.

### Expression and Refolding of rSKTI

The *E. coli* BL21 (DE3) strains were routinely cultivated at 37 °C in Luria-Bertani medium containing 50 μg/mL kanamycin. When the cells reached a optical density (OD600) of 0.9 with UV spectrophotometry, the cells were induced by isopropyl-β-d-thiogalactopyranoside (IPTG) with a final concentration of 0.5 mM. After growing for an additional 4 h at 37 °C, the cells were harvested by centrifugation at 6000 rpm for 20 min and lysed by ultrasonication. Then, inclusion bodies were separated by centrifugation at 12000 rpm for 15 min at 4 °C. Triton X-100 (0.5%, v/v) was used as a detergent to purify the inclusion bodies. The inclusion bodies were washed with 20 mM Tris-HCl buffer (pH 8.0) three times to eliminate Triton X-100.

Purified inclusion bodies were denatured and then diluted in refolding buffer. The final concentration of protein in the refolding buffer was 1 mg/mL. A L25(5^6^) orthogonal experiment design was adopted to screen three key refolding factors (Table 1). The optimum level for each factor was obtained according to the activity of rSKTI after refolding. Single-factor experiment was further optimized to achieve the higher yield.

**Table 1:** Orthogonal design of rSKTI refolding condition

Levels	Factors		
	pH	Temperature (°C)	Redox couple (GSH+GSSG, mM)
1	8.5	4	0+0
2	9.0	10	1+0
3	9.5	16	1+0.125
4	10.0	23	1+0.25
5	10.5	30	4+1

### Purification of rSKTI

The refolded rSKTI was purified by DEAE-FF anion-exchange chromatography (Best Chrom, China). The activated rSKTI was eluted from the column by a linear 0–500 mM NaCl gradient in 20 mM Tris-HCl, pH 8.0. The samples with activity were pooled and stored at −20 °C for further study.

### SKTI Activity Assay

SKTI activity assay method is based on the United States Pharmacopoeia (USP) with a little modification [15]; its activity against trypsin was assayed based on the activity difference of trypsin in the absence or presence of SKTI. The trypsin activity from the positive control group (without SKTI) and the experiment group (with SKTI) was measured in 67 mM phosphate buffer (PB, pH 7.6) at 25 °C with BAEE as substrate. Here, the positive control group consisted of 50 μL 1.35 mg/mL trypsin and 950 μL 67 mM PB, pH 7.6. The experiment group consisted of 50 μL 1.35 mg/mL trypsin, 50 μL 0.63 mg/mL SKTI, and 900 μL 67 mM PB, pH 7.6. One trypsin inhibitor unit (EPU) is defined that will decrease the activity of two trypsin units by 50% where one trypsin unit is defined that will hydrolyze 1.0 μmol of BAEE per second in pH 7.6 at 25 °C. The activity of SKTI was calculated as follows.

1 $$ \mathrm{Activity}\kern0.33em \mathrm{of}\kern0.33em \mathrm{SKTI}\kern0.33em \left(\mathrm{EPU}/\mathrm{L}\right)=\frac{\varDelta {\mathrm{A}}_{253,\kern0.33em \mathrm{U}0}-\varDelta {\mathrm{A}}_{253,\kern0.33em \mathrm{U}1}}{0.001\times 270\times 60\times \mathrm{t}\times \mathrm{V}}\times \mathrm{df}\times 1000 $$

where Δ*A*253,U0 and Δ*A*253,U1 are the change of absorbance at 253 nm within schedule time from the positive control group and the experiment group, respectively; *t* is the reaction time, 3 min; *V* is the volume of reaction solution, 100 μL; 0.001 is the change in absorbance, corresponding to one trypsin unit; 270 is conversion coefficient of FIP unit, one FIP unit is equal to 270 BAEE units; 60 is conversion coefficient of EPU unit, one EPU unit is equal to 60 FIP units; df is dilution factor of SKTI in reaction solution, 20; and 1000 is the conversion coefficient of volume unit.

Protein content was measured according to the BCA method [16], using bovine serum albumin as the standard protein. All assays were performed in triplicate.

### Biochemical Properties

#### Effects of Temperature on Activities and Stabilities of SKTI and rSKTI

The effects of temperature on the activities of SKTI and rSKTI were investigated. Both the positive control group U0 (1.35 mg/mL trypsin in 67 mM PB, pH 7.6.) and the experiment group U1 (1.35 mg/mL trypsin plus 0.63 mg/mL SKTI in 67 mM PB, pH 7.6) were incubated in a different temperature, ranging from 4 to 65 °C and then kept for 2 h. The remained trypsin activity was measured and inhibitory activity of SKTI was calculated as described in “SKTI Activity Assay.” The inhibition rate was calculated based on the following equation. The results are expressed as the mean value ± S.D. of triplicate assay.

2 $$ \mathrm{Inhibition}\kern0.33em \mathrm{rate}=\left(1-\mathrm{U}1/\mathrm{U}0\right)\times 100\% $$

where U0 and U1 are the activity of trypsin in the positive control group and the experiment group, respectively.

For the thermal stability of SKTI and rSKTI, 0.63 mg/mL SKTI or rSKTI in 67 mM PB, pH 7.6) was incubated in a different temperature as above. The trypsin activity was measured and inhibitory activity of SKTI was calculated as described in “SKTI Activity Assay.” The results are expressed as the mean value ± S.D. of triplicate assay.

#### Effects of pH on Activities and Stabilities of SKTI and rSKTI

The effects of pH on the activities of SKTI and rSKTI were investigated. Both the positive control group U0 (1.35 mg/mL trypsin) and the experiment group U1 (1.35 mg/mL trypsin plus 0.63 mg/mL SKTI) were prepared in different pH buffers, including 100 mM HAc-NaAc (pH 3.0–6.0), 100 mM Tris-HCl (pH 7.0–8.0) and 100 mM Gly-NaOH (pH 9.0–11.0), and then kept at 25 °C for 12 h. The remained trypsin activity was measured and inhibitory activity of SKTI was calculated as described in “SKTI Activity Assay.” The inhibition rate was calculated based on the Eq. (2). The results are expressed as the mean value ± S.D. of triplicate assay.

For the pH stability of SKTI and rSKTI, both 0.63 mg/mL SKTI and rSKTI were incubated in above buffer solutions at 25 °C for 12 h. The trypsin activity was measured and inhibitory activity of SKTI was calculated as described in “SKTI Activity Assay.” The results are expressed as the mean value ± S.D. of triplicate assay.

#### Effects of Metal Ions and Organic Solvents on Stabilities of SKTI and rSKTI

The concentration of 100 mM various types of metal ion solutions was prepared including Ca^2+^, Ba^2+^, Mg^2+^, Co^2+^, Cu^2+^, Mn^2+^, Ni^2+^, Fe^3+^, Zn^2+^, and Al^3+^. Both 0.63 mg/mL SKTI and rSKTI were prepared in 67 mM Tris-HCl buffer (pH 7.6) with above metal ion solutions at the final concentration of 1 mM at 25 °C for 4 h.

Both SKTI and rSKTI (0.63 mg/mL) were prepared with various types of organic solvents at the final concentration of 10% (v/v), including dimethyl sulfoxide (DMSO), methanol (MeOH), ethanol (EtOH), acetonitrile (ACN), glycerin, acetone, epoxy chloropropane (EPI), diisopropyl ether (DIPE), and isoamyl alcohol (IAOH), and kept at 25 °C for 4 h.

The trypsin activity was measured and inhibitory activity of SKTI was calculated as described in “SKTI Activity Assay.” The only difference is that 67 mM PB buffer (pH 7.6) is substituted with 67 mM Tris-HCl buffer (pH 7.6) for activity assay. The results are expressed as the mean value ± S.D. of triplicate assay.

### Kinetic Parameter Assay

The kinetic parameters including Michaelis constant (*K*m) and maximal velocity (*V*_max_) were determined based on the Michaelis-Menten equation by Lineweaver-Burk method in the presence and absence of inhibitor. The various substrate concentrations of BAEE was 0.01 mM, 0.015 mM, 0.02 mM, 0.03 mM, 0.05 mM, and 0.075 mM. All assays were performed in triplicate at 25 °C in 67 mM PB (pH 7.6). The value of the inhibition constant (*K*I) was calculated by the following equation:

3 $$ \raisebox{1ex}{${V}_{\mathrm{max}}$}\!\left/ \!\raisebox{-1ex}{${V}_{\mathrm{max}}^{\prime }$}\right.=1+\raisebox{1ex}{$\left[I\right]$}\!\left/ \!\raisebox{-1ex}{${K}_I$}\right. $$

where *V*_max_ and *V*′_max_ are the maximum of activated trypsin activity in the absence and presence of inhibitor, respectively; [*I*] is the concentration of inhibitor; and *K*I is the inhibition constant.

### Molecular Modeling

SWISS-MODEL (http://swissmodel.expasy.org/) was used for homologous modeling of the SKTI and trypsin. AutoDock Vina (http://vina.scripps.edu/) was used to protein-protein molecular docking and screens the feasible results. All PDB files of protein structures were visualized on PyMol (http://pymol.sourceforge.net/).

## Results

### Expression of rSKTI

**Fig. 2:**
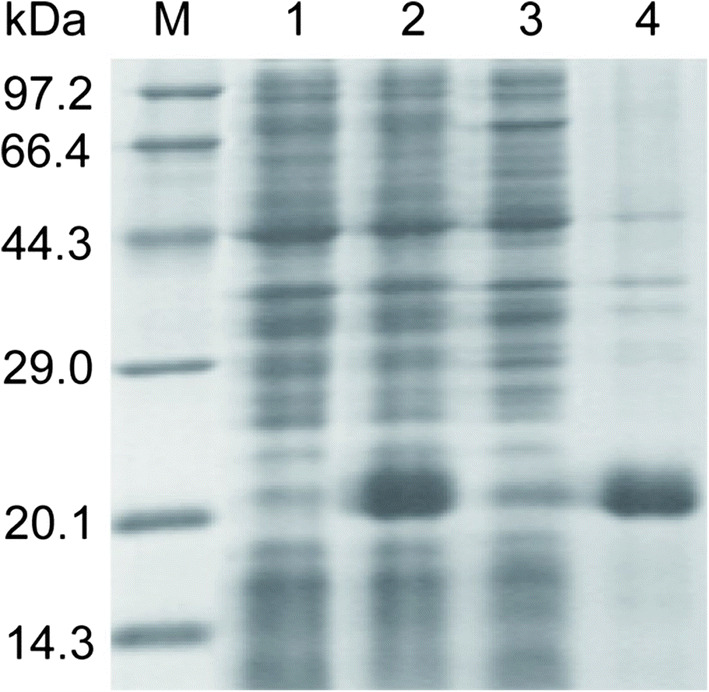
Fifteen percent of SDS-PAGE analysis of the expression of rSKTI from *E. coli*. Line M, molecular weight marker; line 1, cell lysate before induction; line 2, cell lysate after 0.5 mM IPTG induction; line 3, soluble protein; line 4, insoluble protein

SDS-PAGE analysis of the expression of rSKTI in *E. coli* BL21 (DE3) showed a significant protein band of about 20.1 kDa, corresponding to the theoretical molecular weight of rSKTI (Fig. 2). In the analysis on soluble and insoluble parts after cell disruption with ultrasonication, the aimed protein band was mainly appeared in insoluble part, indicating it was expressed as inclusion body (Fig. 2, line 4).

### Optimizing Refolding of rSKTI

**Fig. 3:**
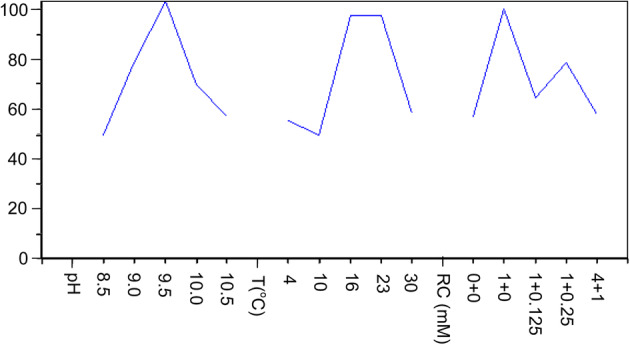
Effects of pH, temperature (T) and redox couple (RC, GSH + GSSG) on the activity of rSKTI in refolding buffer. Ten milligrams per milliliter of rSKTI inclusion bodies (wet weight) was dissolved in denaturating buffer and then diluted in refolding buffer (1:10 ratio)

Several key factors that influenced the refolding such as pH, temperature, and redox couples were selected for orthogonal experiment. The analysis of effect of each factor on refolding is shown in Fig. 3, which is based on 25 separated groups of orthogonal design. The optimal condition for rSKTI refolding was pH 9.5, 16 °C, 1 mM GSH and pH 9.5, 23 °C, 1 mM GSH.

**Fig. 4:**
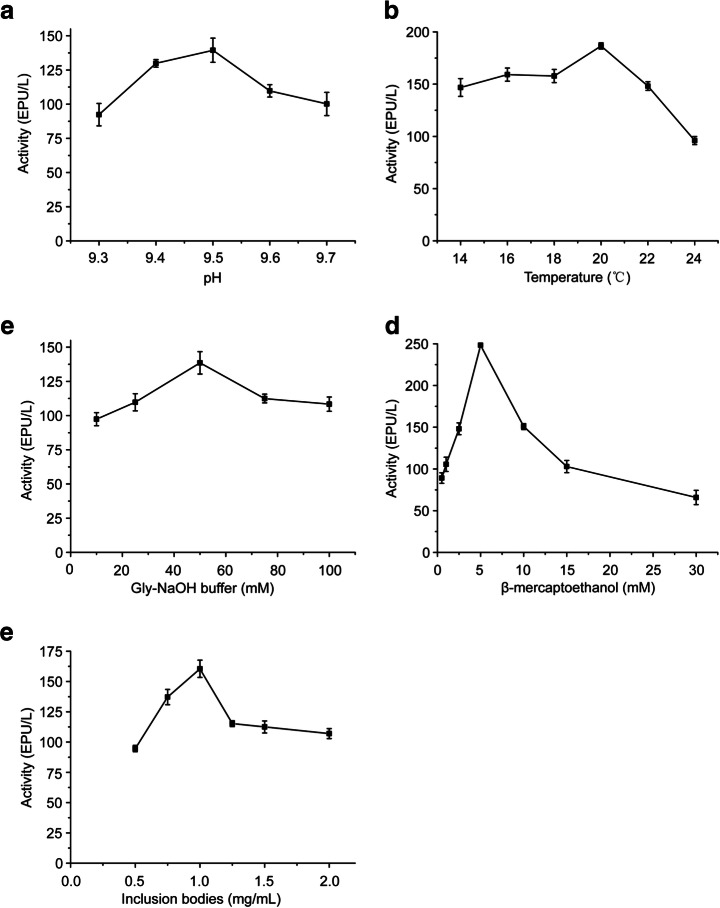
Optimization of various factors on rSKTI refolding. **a** pH. **b** Temperature. **c** Concentration of Gly-NaOH buffer. **d** Concentration of β-mercaptoethanol. **e** Contents of inclusion bodies. The results of each series were expressed as the mean ± S.D. of triplicate assays

Based on the orthogonal results, a more detailed single-factor experiment was designed to further optimize the refolding condition, such as refolding buffer pH, temperature, and Gly-NaOH buffer concentration. The results are shown in Fig. 4 a, b, and c, respectively. Considering that the denature condition would have effects on its refolding recovery, the key factors were investigated, such as concentration of β-mercaptoethanol and inclusion body in denature buffer. The results are shown in Fig. 4 d and e.

Taken these, a final condition was established for rSKTI unfolding and refolding. The rSKTI inclusion bodies (10 mg/mL) was dissolved in denaturation buffer (50 mM Gly-NaOH, 8 M urea, 10 mM EDTA, and 5 mM β-mercaptoethanol, pH 9.5) for 2 h and then diluted in refolding buffer (50 mM Gly-NaOH, 1 mM EDTA, and 1 mM GSH, pH 9.5) with a ratio of 1:10 (v:v) at 20 °C for 20 h.

### Purification of rSKTI

**Fig. 5:**
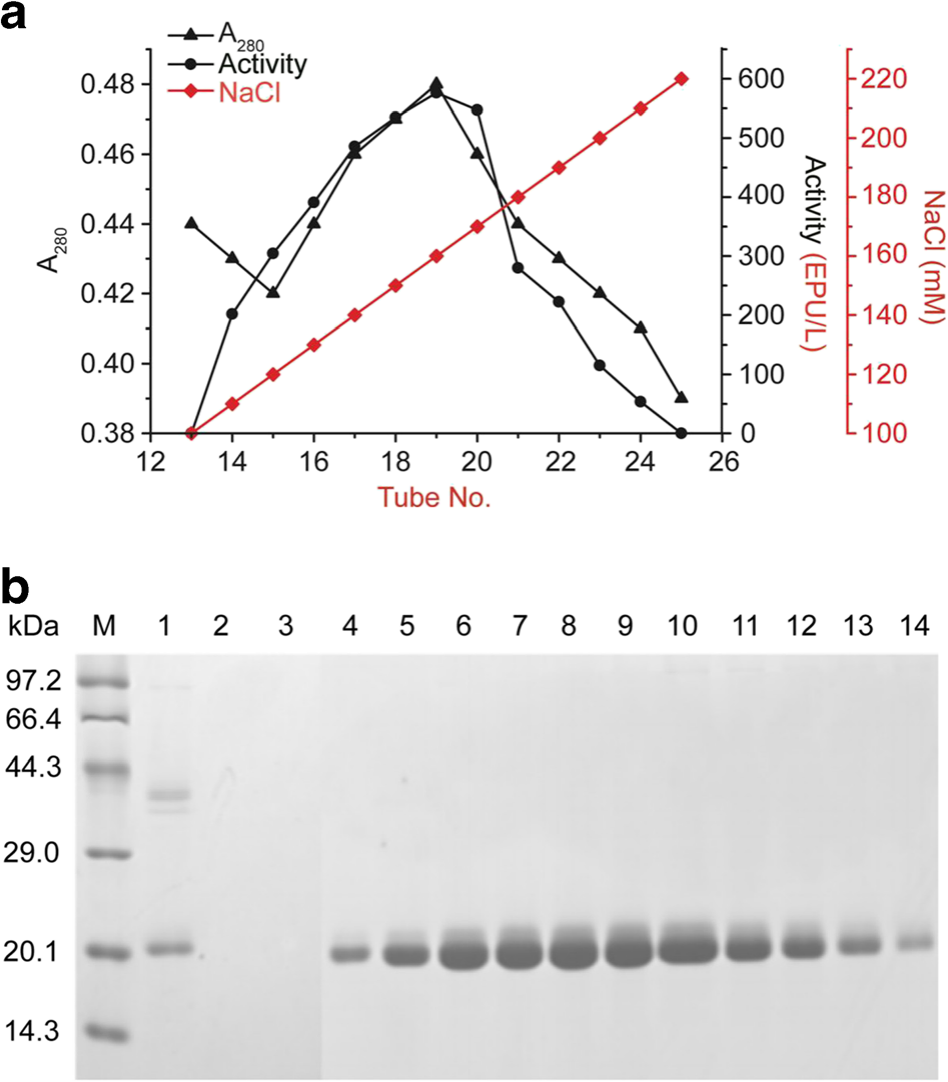
Purification of rSKTI with DEAE-FF anion-exchange chromatography. **a** The elution curve of rSKTI. The sample was eluted with a linear gradient of 0–500 mM NaCl in 20 mM Tris-HCl buffer (pH 8.0). **b** Fifteen percent of SDS-PAGE analysis of eluate samples. Lane M, molecular weight marker; lane 1, loaded sample; lane 2, flow through sample when loading; lane 3, flow through sample at equilibrium; lane 4~14, the purified rSKTI protein

DEAE-FF anion-exchange chromatography was used to concentrate and purify active rSKTI successfully. The eluents were highly pure, as shown by 15% SDS-PAGE analysis (Fig. 5). The final activity yield of rSKTI achieved 70%, and the specific activity of rSKTI was improved over three fold after purification. All data are summarized in Table 2. The samples from tube no. 14 to tube no. 24 with activity were pooled and concentrated with ultrafiltration. The rSKTI was 1.9 mg/mL after concentration.

**Table 2:** Summary of rSKTI purification

Step	Volume (mL)	Total activity	Total protein	Specific activity	Yield of protein	Yield of activity
		(EPU)	(mg)	(EPU/mg pro)	(%)	(%)
Before purification	200	53.50	100.45	0.53	100	100
After purification	110	37.13	30.95	1.68	30.81	69.40

### RSKTI Activity Assay

**Fig. 6:**
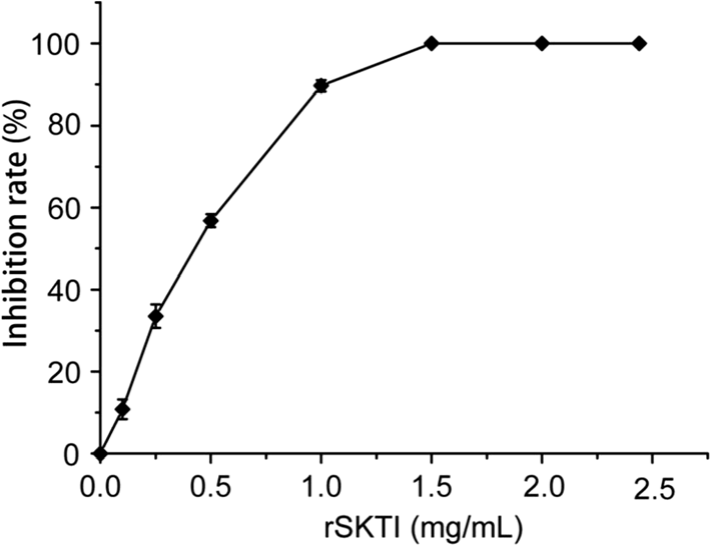
Inhibitory effect of rSKTI against trypsin. Trypsin (1.35 mg/mL) was inhibited by increasing the contents of rSKTI. The value of 100% activity refers to trypsin activity without rSKTI. Each value represents an experiment performed in a triplicate (mean ± S.D.)

IC50 value, defined as the amount of rSKTI when half trypsin activity was inhibited, was about 0.5 mg/mL. Moreover, the trypsin activity was completely inhibited by rSKTI at the concentration of 1.5 mg/mL (Fig. 6). There existed a stable binary complex formed between rSKTI and trypsin with equimolar amount based on the concentration of trypsin and rSKTI.

### Biochemical Properties

#### Effects of Temperature on Activities of SKTI and rSKTI

**Fig. 7:**
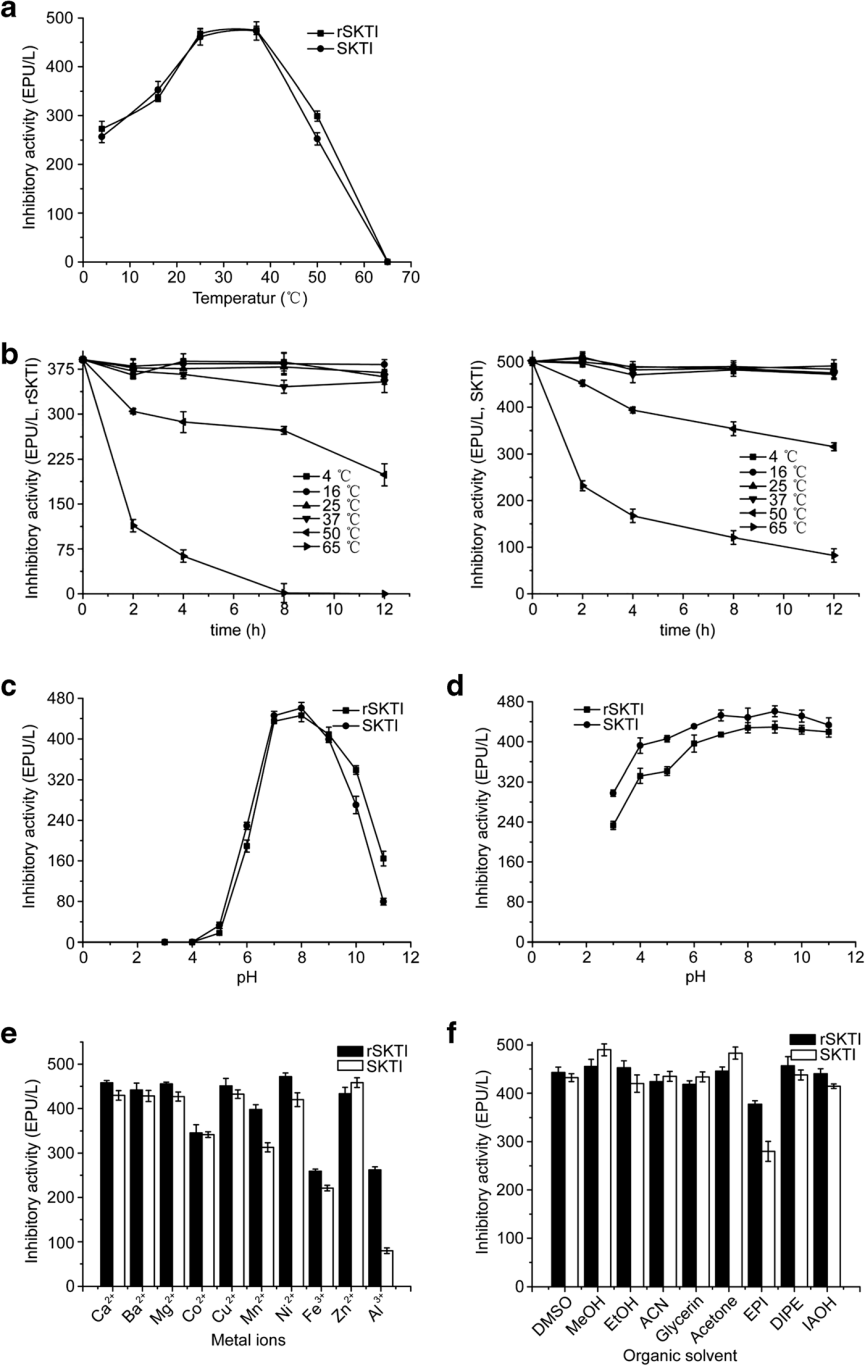
Biochemical properties of SKTI and rSKTI. **a** Optimal temperature. **b** Thermal stability. **c** Optimal pH. **d** pH stability. **e** Metal ions. **f** Organic solvents. The results of each series were expressed as the mean±S.D of triplicate assays

When temperature rose from 4 to 65 °C, the activity of trypsin from positive control group (trypsin without SKTI or rSKTI) increased followed by decreased and reached the maximum at 37 °C. Meanwhile, the activity of trypsin from treatment group (trypsin containing SKTI or rSKTI) was almost consistent. The optimum temperature of inhibitor including SKTI and wild type was 35 °C (Fig. 7a). It was noticeable that both SKTI and wild type have completely lost activity against trypsin when temperature was over 65 °C.

#### Thermal Stability of SKTI and rSKTI

The SKTI and rSKTI were quite stable below 37 °C (Fig. 7b). After incubation for 12 h at 50 °C, rSKTI retained 50% of its activity. In contrast, SKTI retained more than 60% of its activity. After incubation for 12 h at 65 °C, rSKTI has completely lost activity against trypsin and SKTI merely retained approximately 15% of its original activity. There were not significant different between the stabilities of SKTI and rSKTI.

#### Effects of pH on Activities of SKTI and rSKTI

Using BAEE as the substrate, the optimal pH was determined to be pH 8.0 for SKTI and rSKTI (Fig. 7c). With the increase of pH, the inhibitory activity of rSKTI was rose followed by declined and reached the maximum at pH 8.0. In acid condition such as below pH 4.0, rSKTI showed no activity against trypsin. The rSKTI could not bind to trypsin because the side chain of Asp189 residue in active center of trypsin was protonized. Almost, the same tendency and the same optimum pH of SKTI were obtained.

#### pH Stability of SKTI and rSKTI

The rSKTI was stable in pH 7.0–11.0; above 90% activities were kept, while in pH 3.0 and pH 4.0 buffers, 50% and 30% activity was lost, respectively. Therefore, we concluded that rSKTI was stable in neutral and alkaline conditions (Fig. 7d). At the same time, the same tendency of pH stabilities of SKTI was obtained.

#### Effects of Metal Ions and Organic Solvents on Stabilities of SKTI and rSKTI

We observed that the activity of inhibitor against trypsin-like was inhibited by Co^2+^, Mn^2+^, Fe^3+^, and Al^3+^ , while it was less affected by other metal ions (Fig. 7e). The activity of rSKTI was reduced to 80% by Co^2+^, 85% by Mn^2+^, and 60% by Fe^3+^ and Al^3+^. Similarly, the activity of wild type was reduced to 85% by Co^2+^, 75% by Mn^2+^, 65% by Fe^3+^, and 30% by Al^3+^. The Co^2+^ and Mn^2+^, which belonged to heavy metal ions, made protein denatured. The Fe^3+^ made protein oxidated with extremely strong oxidability. The Al^3+^ was easily combined with protein, affecting the catalytic reactions involved in the enzyme. In general, organic solvents had little effect on the activity of inhibitor (SKTI and rSKTI), which might be that the hydrolysis reaction of trypsin mainly occurred in aqueous phase instead of in organic phase (Fig. 7f). However, the activity of rSKTI and SKTI was reduced to 85% and 75% by epichlorohydrin, respectively.

### Kinetic Parameter Assay

**Fig. 8:**
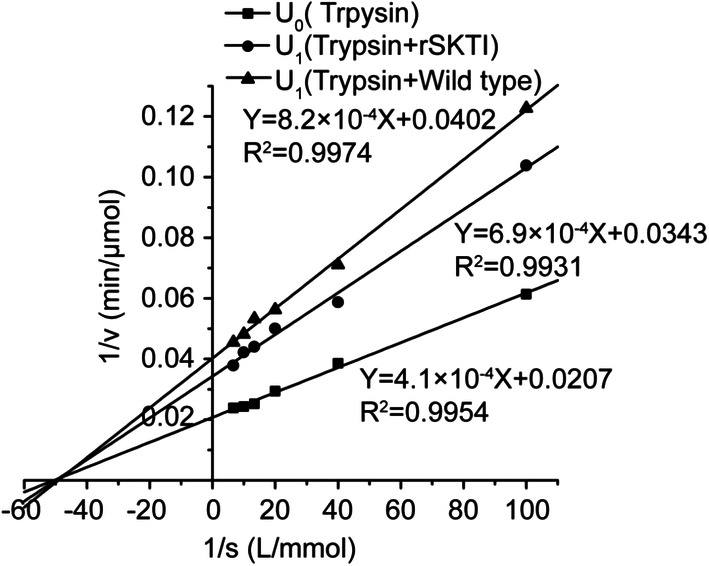
Lineweaver-Burk plots analysis of the inhibition kinetics. Assay of the trypsin (1.35 mg/mL) activity in the presence and absence of SKTI (0.63 mg/mL) or rSKTI (0.63 mg/mL)

Kinetic parameters of trypsin in the presence and absence of inhibitors (SKTI or rSKTI) were determined, and the results are shown in Fig. 8. The *K*m values of three groups were same, while the *V*_max_ values of three groups were different (Table 3). This indicated that the incorporation of SKTI to trypsin did not alter the affinity between trypsin and substrate but reduced the catalytic reaction rate of trypsin. It could be induced that the inhibition type was belong to a non-competitive inhibition. The *K*I value was calculated as 2.2 μM and 1.67 μM for rSKTI and SKTI, respectively. The specific activity of rSKTI was 0.71 EPU/mg pro, lower than that of SKTI (0.85 U/mg pro). Perhaps, higher purity for rSKTI attributed to a lower *K*I value compared with SKTI.

**Table 3:** Kinetic parameters of trypsin in various conditions

Group	*K*_m_ (mM)	*V*_max_ (μmol/min)
Trypsin (U0)	0.02	48.31
Trypsin + rSKTI (U1)	0.02	29.15
Trypsin + SKTI (U1)	0.02	24.87

## Discussion

In this study, the results of inhibition kinetic assay as Lineweaver-Burk plots analysis of rSKTI against trypsin showed an unchanged *K*m and decreased *V*_max_ value of trypsin. The inhibition mechanism of rSKTI against trypsin might be non-competitive even if more amounts of substrates existed. In addition, the *K*I value of rSKTI was 2.29 μM, which was far less than *K*m value of trypsin that was 0.02 mM. All above suggested that the inhibition of rSKTI to trypsin was more likely to be irreversible type instead of non-competitive type. It was contrary to the previous report that the SKTI was a competitive inhibitor to trypsin [17].

**Fig. 9:**
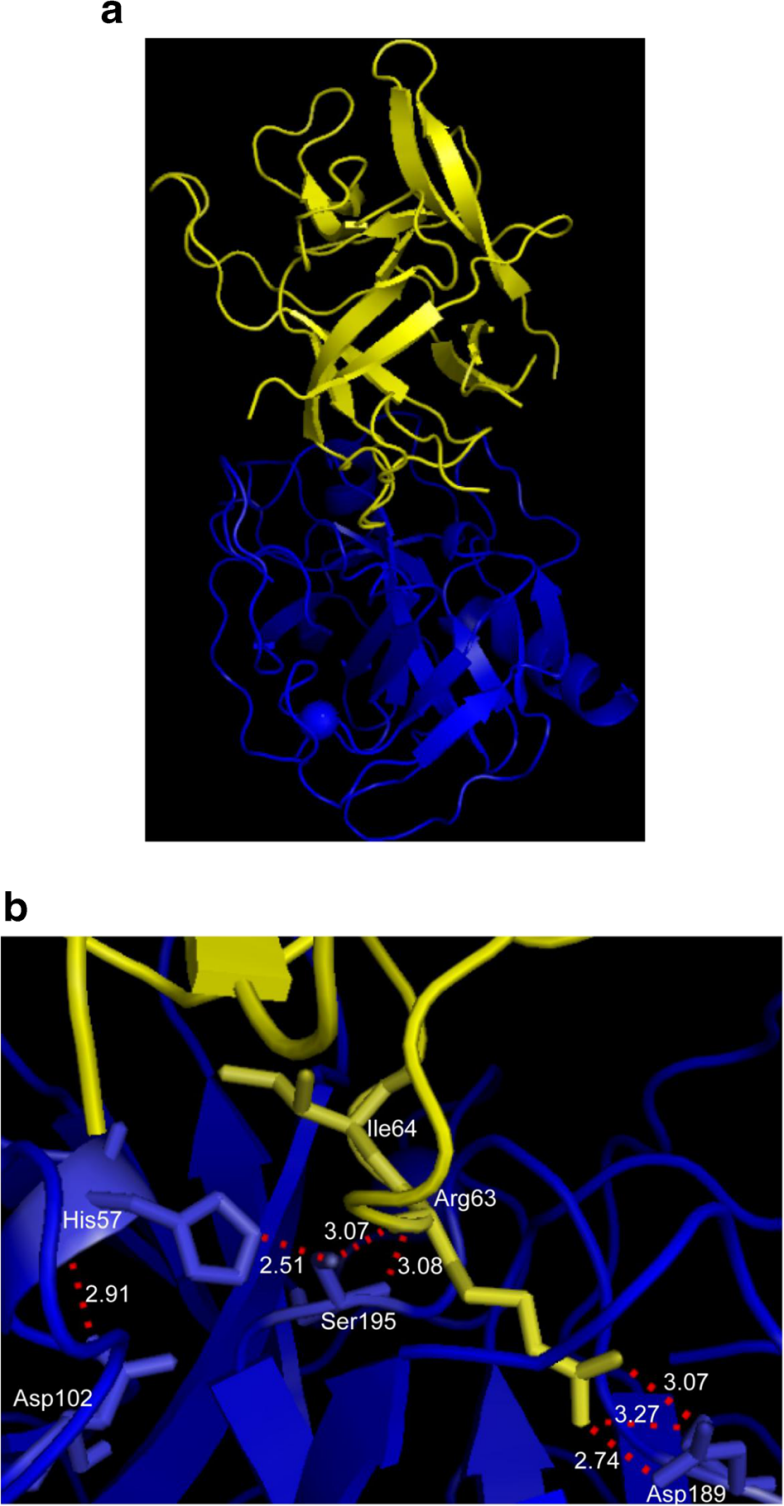
Three-dimensional model of interaction SKTI (yellow) with trypsin (blue). **a** Visualization of the binary complex in SKTI-trypsin. **b** The distance of hydrogen bonds formed by the residues in active region. The inhibitor and proteinase backbone is shown as cartoon. Sticks indicate residues involved in enzyme-inhibitor interaction

For the inhibition mechanism between inhibitor and enzyme, it was generally accepted that the side chain of Arg63 residue in SKTI carried the positive charges, forming strong electrostatic interaction with the negative charges of the side chain of Asp189 in trypsin, significantly contributing to the binding of inhibitor to the active center of trypsin [18, 19]. Here, three-dimensional structure of compound is shown in Fig. 9. It was interesting to note that SKTI formed a binary complex with trypsin at the ratio of 1:1 (mol:mol), which was confirmed by trypsin inhibition assay in vitro. Figure 9 b highlights the interaction between SKTI and trypsin in detail with molecular docking technology. We observed that there were five interactions between Arg63 of SKTI and active domain of trypsin, including three catalytic sites (His57, Asp102, and Ser195) and one binding site (Asp189). It successfully blocked the active center of trypsin and effectively prevented the binding of substrates to trypsin. The side chain guanidine group of Arg63 in SKTI was hydrogen-bonded to the side chain carboxyl group of Asp189 in trypsin, with distance of 2.74 Å, 3.07 Å, and 3.27 Å, respectively. In addition, the side-chain hydroxyl group of Ser195 in trypsin acted as a proton donor, while the main chain NH of Arg63 in SKTI acted as a related receptor, at a distance of 3.07 Å. The main NH of Ser195 from trypsin interacted with the main CO of Arg63 in SKTI, forming a hydrogen bond of 3.08 Å.

Combining above three-dimensional structure analysis with inhibition kinetics behavior gave evidences that the inhibition mechanism of SKTI to trypsin should be irreversible. The fact that *K*I value of inhibitor was far less than *K*m value of enzyme suggested that SKTI had better affinity and more easily bound to trypsin compared with substrate. Rühlmann et al. [20] found that there was a covalent bond between bovine pancreatic trypsin inhibitor (BPTI) and trypsin based on the electron density at the active region of tetrahedral intermediate, whereas the peptide bond of Lys15-Ala16 at the active site of BPTI was still linked. Later, the research [21] discovered that the scissile peptide bond of SKTI between Arg63 and Ile64 was cleaved by trypsin, because it could specifically digest the peptide bond formed by alkaline amino acid (lysine and arginine) at carboxyl terminus. According to the catalytic mechanism of trypsin to SKTI was analogous to synthetic substrate (BAEE), we further speculated that SKTI was the part of Kcat type inhibitors in terms of trypsin. Of course, further study was needed to validate the hypothesis.

## Conclusion

In this research, an efficient method was established to produce rSKTI in *E. coli* with optimized refolding technology. Biochemical properties of rSKTI studied here should be useful for the better application of rSKTI in production. Furthermore, the specific study on inhibition kinetic behavior and molecular structure modeling of complex gave new insight into inhibition mechanism of SKTI against trypsin. These results provide reference to further research on the inhibition of other Kunitz trypsin inhibitors to their target protease.

## Electronic Supplementary Material

ESM 1 (DOCX 52 kb)
