# Supplementary material for: Preparation and Irreversible Inhibition Mechanism Insight into a Recombinant Kunitz Trypsin Inhibitor from Glycine max L. Seeds
Source: Appl Biochem Biotechnol. 2020 Feb 1;191(3):1207–22. doi: 10.1007/s12010-020-03254-5 (PMC7320042; doi:10.1007/s12010-020-03254-5)
Supplement: Supplementary file 1 — (DOCX 52 kb) [file 12010_2020_3254_MOESM1_ESM.docx]

Table S1 The results of orthogonal design

|  | A | B | C | (EPU/L) |
| --- | --- | --- | --- | --- |
| 1 | 1 | 1 | 1 | 42.98 |
| 2 | 1 | 2 | 2 | 41.61 |
| 3 | 1 | 3 | 3 | 90.53 |
| 4 | 1 | 4 | 4 | 54.87 |
| 5 | 1 | 5 | 5 | 16.92 |
| 6 | 2 | 1 | 3 | 35.67 |
| 7 | 2 | 2 | 4 | 79.10 |
| 8 | 2 | 3 | 5 | 79.10 |
| 9 | 2 | 4 | 1 | 117.28 |
| 10 | 2 | 5 | 2 | 81.85 |
| 11 | 3 | 1 | 5 | 40.24 |
| 12 | 3 | 2 | 1 | 32.01 |
| 13 | 3 | 3 | 2 | 216.73 |
| 14 | 3 | 4 | 3 | 112.48 |
| 15 | 3 | 5 | 4 | 113.85 |
| 16 | 4 | 1 | 2 | 69.96 |
| 17 | 4 | 2 | 3 | 55.33 |
| 18 | 4 | 3 | 4 | 58.53 |
| 19 | 4 | 4 | 5 | 112.94 |
| 20 | 4 | 5 | 1 | 52.13 |
| 21 | 5 | 1 | 4 | 86.42 |
| 22 | 5 | 2 | 5 | 39.32 |
| 23 | 5 | 3 | 1 | 41.61 |
| 24 | 5 | 4 | 2 | 90.08 |
| 25 | 5 | 5 | 3 | 27.43 |
| T1 | 246.91 | 275.27 | 286.03 |  |
| T2 | 393.02 | 247.37 | 500.20 |  |
| T3 | 515.35 | 486.47 | 321.46 | 1788.99 |
| T4 | 348.85 | 487.65 | 392.82 |  |
| T5 | 284.86 | 292.23 | 288.48 |  |
| ↓x1 | 49.38 | 55.05 | 57.21 |  |
| ↓x2 | 78.60 | 49.47 | 100.04 |  |
| ↓x3 | 103.07 | 97.29 | 64.29 |  |
| ↓x4 | 69.77 | 97.53 | 78.56 |  |
| ↓x5 | 56.97 | 58.45 | 57.70 |  |
| R | 53.69 | 48.06 | 42.83 |  |

No Factors Activity*

*The results of each series were expressed as the mean of triplicate assays.

Table S2 Effects of temperature on activity of rSKTI Trypsin activity (BAEE units/mL) a Inhibition rate b

Activity of

| Temperature Trypsin Trypsin + rSKTI | | | (%) | rSKTI |
| --- | --- | --- | --- | --- |
| (℃) | (U0) | (U1) |  | (EPU/L) |
| 4 | 6911.11±167.77 | 2488.89±252.39 | 63.99 | 272.98±15.58 |
| 16 | 8044.44±203.67 | 2600.01±115.47 | 67.68 | 336.08±7.13 |
| 25 | 11044.44±76.98 | 3466.67±176.38 | 68.61 | 467.76±10.89 |
| 37 | 12111.11±177.56 | 4422.22±138.78 | 63.49 | 474.62±8.57 |
| 50 | 7733.33±176.38 | 2888.89±167.77 | 62.64 | 299.04±10.36 |
| 65 | 577.78±101.84 | 1422.22±138.78 | 0 | 0 |

a Both the positive control group U0 (1.35 mg/mL trypsin in 67 mM PB, pH 7.6.) and the experiment group U1 (1.35 mg/mL trypsin plus 0.63 mg/mL rSKTI in 67 mM PB, pH 7.6) were

incubated in different emperature, ranging from 4 ℃ to 65 ℃, for 2 h.

b Inhibition rate = (U0-U1/U0)*100%.

Table S3 Effects of temperature on activity of SKTI Trypsin activity (BAEE units/mL) a Inhibition rate b

Activity of

Temperature

(℃)

|  | (U0) | (U1) |  | (EPU/L) |
| --- | --- | --- | --- | --- |
| 4 | 7600.00±176.38 | 3444.44±192.45 | 54.68 | 256.52±11.88 |
| 16 | 10088.89±335.55 | 4377.78±277.56 | 56.61 | 352.54±17.13 |
| 25 | 12288.89±269.43 | 4822.22±269.43 | 60.76 | 460.91±16.63 |
| 37 | 13666.67±240.37 | 6000.00±305.51 | 56.10 | 473.25±18.86 |
| 50 | 8244.44±252.39 | 4155.56±203.67 | 49.59 | 252.40±12.57 |
| 65 | 466.67±66.67 | 1066.67±66.67 | 0 | 0 |

Trypsin

Trypsin + SKTI

(%)

SKTI

a Both the positive control group U0 (1.35 mg/mL trypsin in 67 mM PB, pH 7.6.) and the experiment group U1 (1.35 mg/mL trypsin plus 0.63 mg/mL rSKTI in 67 mM PB, pH 7.6) were incubated in different emperature, ranging from 4 ℃ to 65 ℃, for 2 h.

b Inhibition rate = (U0-U1/U0)*100%.

Table S4 Effects of pH on activity of rSKTI Trypsin activity (BAEE units/mL) b Inhibition

| pH a | Trypsin | Trypsin+rSKTI | rate c | rSKTI |
| --- | --- | --- | --- | --- |
|  | (U0) | (U1) | (%) | (EPU/L) |
| 3 | 0 | 0 | / | 0 |
| 4 | 0 | 0 | / | 0 |
| 5 | 644.44±153.96 | 355.56±76.98 | 44.83 | 17.83±4.75 |
| 6 | 4711.11±203.67 | 1644.44±192.45 | 65.09 | 189.30±11.88 |
| 7 | 9844.44±300.62 | 2800.00±66.67 | 71.56 | 434.84±4.12 |
| 8 | 10733.33±333.33 | 3666.67±200.00 | 65.84 | 446.21±12.35 |
| 9 | 10533.33±133.33 | 3911.11±234.13 | 62.87 | 408.78±14.45 |
| 10 | 9822.22±234.13 | 4333.33±133.33 | 55.88 | 338.82±8.23 |
| 11 | 7355.56±153.96 | 4688.89±234.13 | 36.25 | 164.61±14.45 |

Activity of

a The various buffers were 100 mM HAc-NaAc (pH 3.0-6.0), 100 mM Tris-HCl (pH 7.0-8.0), 100 mM Gly-NaOH (pH 9.0-11.0).

b Trypsin (1.35 mg/ml) without rSKTI was the positive control group. Trypsin (1.35 mg/ml) mixed rSKTI (0.63 mg/ml) was the treatment group. All groups were prepared in above buffers at 25 ℃ for 2 h.

c Inhibition rate = (U0-U1/U0)*100%.

Table S5 Effects of pH on activity of SKTI Trypsin Activity (BAEE units/mL) b Inhibition

Activity of SKTI

pH a

|  | (U0) | (U1) | (%) |  |
| --- | --- | --- | --- | --- |
| 3 | 0 | 0 | / | 0 |
| 4 | 0 | 0 | / | 0 |
| 5 | 955.56±101.84 | 422.22±101.84 | 55.81 | 32.92±6.29 |
| 6 | 5244.44±252.39 | 1533.33±115.47 | 70.76 | 229.08±7.13 |
| 7 | 9600.00±66.67 | 2377.78±138.78 | 75.23 | 445.82±8.57 |
| 8 | 10800.00±230.94 | 3333.33±176.38 | 69.15 | 460.91±10.89 |
| 9 | 10622.22±138.78 | 4155.56±101.84 | 60.88 | 399.18±6.29 |
| 10 | 9888.89±138.78 | 5511.11±277.56 | 44.27 | 270.23±17.13 |
| 11 | 7133.33±266.67 | 5844.44±269.43 | 18.07 | 79.56±6.63 |

Trypsin

Trypsin+SKTI

rate c

(EPU/L)

a The various buffers were 100 mM HAc-NaAc (pH 3.0-6.0), 100 mM Tris-HCl (pH 7.0-8.0), 100 mM Gly-NaOH (pH 9.0-11.0).

b Trypsin (1.35 mg/ml) without SKTI was the positive control group. Trypsin (1.35 mg/ml) mixed SKTI (0.63 mg/ml) was the treatment group. All groups were prepared in above buffers at 25 ℃ for 2 h.

c Inhibition rate = (U0-U1/U0)*100%.
